# Noninflammatory extrafacial edema as a clue to the diagnosis of vacuoles, E1 enzyme, X-linked, autoinflammatory, somatic (VEXAS) syndrome

**DOI:** 10.1016/j.jdcr.2024.02.016

**Published:** 2024-03-02

**Authors:** Michael J. Diaz, Vivian Y. Liu, Kiran Motaparthi

**Affiliations:** a College of Medicine, University of Florida, Gainesville, Florida; b Department of Dermatology, University of Florida, Gainesville, Florida

**Keywords:** extrafacial edema, myelodysplastic syndrome, periorbital edema, Sweet syndrome, systemic inflammation, treatment-refractory, UBA1 sequencing, ubiquitin-activating enzyme, VEXAS

## Introduction

Vacuoles, E1 enzyme, X-linked, autoinflammatory, somatic (VEXAS) syndrome is a progressive autoinflammatory disease notable for concomitant rheumatologic and hematologic features. This disease was first described in 2020 when somatic mutations were identified in the ubiquitin-activating enzyme 1 (*UBA1*) gene in 25 men with an adult-onset autoinflammatory disorder, often accompanied by myeloid dysplasia and overlapping clinical phenotypes of disparate diagnoses and shared patterns of histopathology.^1^ This mutation in myeloid cells is caused by substitutions of Methionine-41 that promote the expression of the inactive isoform *UBA1c*, resulting in reduced ubiquitylation activity.^2^

VEXAS syndrome represents a new paradigm of how genetic mosaicism can result in adult-onset monogenic autoinflammatory symptoms involving the skin, lungs, blood vessels, and joints.^3^ Cytoplasmic vacuolization is also typically seen in erythroid and myeloid precursors in the bone marrow. Cutaneous manifestations are the presenting features in >60% of VEXAS patients. Most commonly, these include neutrophilic dermatoses, cutaneous vasculitis, and noninflammatory edema (namely periorbital).^4^ The abnormal edema is especially distinctive of VEXAS syndrome and its presence, together with systemic inflammation, serve as clues to the diagnosis.

## Case report

A man in his 50s with a history of myelodysplastic syndrome (MDS), deep vein thrombosis, pulmonary embolism, chronic macrocytic anemia and thrombocytopenia, and intermittent night sweats and fever presented with Sweet syndrome refractory to many therapies. Prior treatments included SSKI, dapsone, cyclosporine, colchicine, anakinra, secukinumab, and rituximab, resulting in either minimal improvement or intolerable side effects. Notably, the patient’s symptoms consistently flared when tapering prednisone below 20 mg/day. The patient had been treated for MDS with 6 cycles of azacytidine and later with decitabine and cedazuridine.

Over the next few years, the patient presented multiple times with scattered erythematous, edematous papules and nodules on the trunk and extremities consistent with flares of Sweet syndrome and refractory to additional treatments including thalidomide and sulfasalazine. At this time, he was still dependent on prednisone.

**Fig 1:**
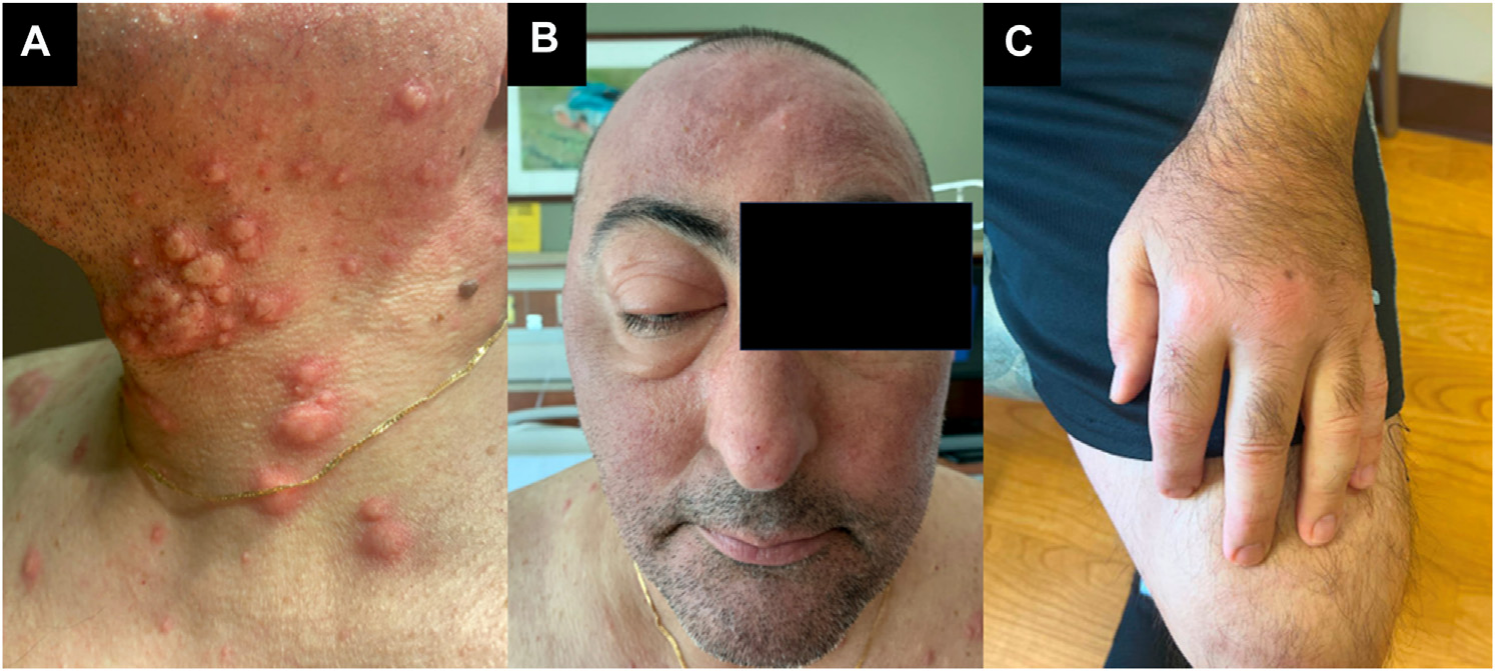
Patient clinical presentation. **A,** Edematous papules and plaques of the neck. **B,** Periorbital edema. **C,** Edema of the hand.

The patient was then admitted to the hospital for periorbital and hand edema as well as edematous papules and plaques on the face, scalp, and trunk, which were initially thought to be due to flaring of Sweet syndrome (Fig 1).

**Fig 2:**
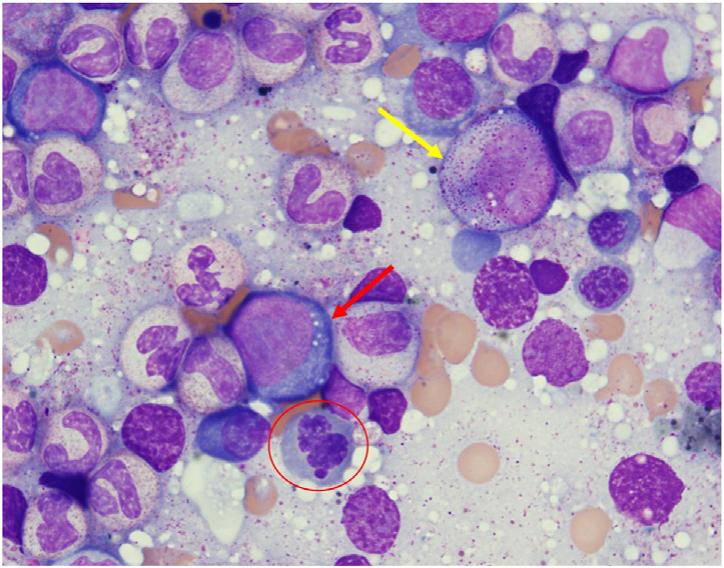
Wright-Giemsa-stained bone marrow smear. Cytoplasmic vacuoles can be seen in scattered myeloid cells (*yellow arrow*) and erythroid cells (*red arrow*). Dyspoietic changes are also noted in erythroid cells, including irregular nuclear contours (*red circle*), consistent with the patient history of myelodysplastic syndrome. Courtesy of Joanna Chaffin, MD.

He was treated with methylprednisolone 60 mg IV with improvement and discharged 2 days later. Over the next year cyclosporine, IVIg, and lenalidomide were trialed but were ineffective as steroid-sparing agents. The refractory nature of the neutrophilic dermatosis coupled with notable, noninflammatory periorbital, and hand edema raised diagnostic suspicion for VEXAS syndrome. Subsequent *UBA1* sequencing of the patient’s bone marrow and skin confirmed the presence of the pathogenic VEXAS mutation in *UBA1*. Bone marrow smear also showed hallmark cytoplasmic vacuolization in myeloid and erythroid cells, corroborating this result (Fig 2).

Prednisone was continued and ruxolitinib was initiated. Even at a dose of 15 mg twice daily, ruxolitinib did not permit tapering of prednisone nor reduce the dependence on transfusions due to progressive anemia and thrombocytopenia. Therefore, the patient received a stem cell transplant, with subsequent resolution of hematologic and cutaneous findings.

## Discussion

VEXAS syndrome is a recently recognized multisystem autoinflammatory syndrome due to somatic *UBA1* mutations in myeloid cells. Skin involvement is often the first symptom for most patients; in a retrospective review of 59 cases of VEXAS syndrome, Sweet syndrome was the initial diagnosis in 47% of patients. Biopsies demonstrated neutrophilic dermatosis in 77% (34/43) of patients.^5^ Sweet syndrome is frequently associated with hematologic malignancies, including MDS. The relationship between Sweet syndrome, hematologic malignancies, and VEXAS syndrome may pose a diagnostic dilemma.

Periorbital edema is present in 5% to 12% of patients with VEXAS, but studies that have described this finding did not specify if the periorbital edema was separate from inflammatory lesions of Sweet.^4^^,^^6^^,^^7^ There are only 2 case reports that specifically discuss periorbital edema as a feature of VEXAS syndrome distinct from lesions of Sweet syndrome.^8^^,^^9^ Compared to Sweet syndrome, the edema in VEXAS is noteworthy because it not only presents within inflammatory lesions, but also manifests as isolated noninflammatory edema.

More significantly, we also identified distinct noninflammatory extrafacial (hand) edema in our patient, which has not been previously described in the literature. This novel clinical clue, in combination with treatment-refractory systemic disease and edema elsewhere, should prompt *UBA1* somatic sequencing.

Differences in treatment response may also help differentiate Sweet and VEXAS syndrome. Typically, Sweet syndrome demonstrates an excellent response to systemic corticosteroids. VEXAS syndrome, on the other hand, does not respond as well to low-dose corticosteroids, and many patients are unable to maintain response to doses of prednisone below 10-15 mg/day. Disease-modifying antirheumatic drugs, tumor necrosis inhibitors, and tocilizumab have also shown only modest benefit.^6^ A retrospective study of Janus kinase signal transducer and activator of transcription inhibitors demonstrated better efficacy with ruxolitinib in the treatment of VEXAS syndrome compared to other Janus kinase signal transducer and activator of transcription inhibitors.^10^ Currently, stem cell transplant is the only treatment with curative potential.^6^

Noninflammatory and extrafacial edema, in association with a recalcitrant neutrophilic dermatosis should prompt diagnostic consideration for VEXAS syndrome.

## Conflicts of interest

None disclosed.
